# Combined effect of silica dust exposure and cigarette smoking on total and cause-specific mortality in iron miners: a cohort study

**DOI:** 10.1186/s12940-018-0391-0

**Published:** 2018-05-09

**Authors:** Hanpeng Lai, Yuewei Liu, Min Zhou, Tingming Shi, Yun Zhou, Shaofan Weng, Weihong Chen

**Affiliations:** 1 Department of Occupational and Environmental Health, School of Public Health, Tongji Medical College, Huazhong University of Science and Technology, 13 Hangkong Road, Wuhan, 430030 Hubei China; 2 Key Laboratory of Environment and Health, Ministry of Education and Ministry of Environmental Protection, and State Key Laboratory of Environmental Health (Incubating), School of Public Health, Tongji Medical College, Huazhong University of Science and Technology, Wuhan, 430030 Hubei China; 3 Hubei Provincial Key Laboratory for Applied Toxicology, Hubei Provincial Center for Disease Control and Prevention, Wuhan, 430079 Hubei China; 4 Shenzhen Prevention and Treatment Center for Occupational Disease, Shenzhen, Guangdong China

**Keywords:** Cohort, Mortality, Silica, Cigarette smoking, Joint effect

## Abstract

**Background:**

Both cigarette smoking and long-term exposure to crystalline silica dust were reported to be associated with increased mortality. However, the combined effect of both factors has not been well evaluated.

**Methods:**

We investigated a retro-prospective cohort of 7,665 workers from one Chinese iron mine with a median follow-up of 42.8 years. Cumulative silica exposure was estimated for each worker by linking work histories with a job-exposure matrix. Cigarette smoking information was collected through face-to-face questionnaires. Hazard ratios (HRs) for total and cause-specific mortality due to silica exposure and smoking were estimated using Cox proportional hazards models.

**Results:**

A total of 2,814 deaths occurred during 315,772.9 person-years of follow-up. Significantly elevated mortality from all causes, cardiovascular disease, non-malignant respiratory disease and lung cancer was observed among silica-exposed workers, while elevated mortality from non-malignant respiratory disease and lung cancer was observed among smokers. Combined exposure to silica dust and cigarette smoking elevated the proportion of mortality and accounted for 21.2, 76.0, 35.7 and 81.4% of all causes, non-malignant respiratory disease, cardiovascular disease, and lung cancer, respectively. Significant additive joint effects of silica exposure and cigarette smoking on mortality from lung cancer (HR 1.893, 95% CI 0.628 to 3.441) and pneumoconiosis (6.457, 0.725 to 39.114), together with a significant multiplicative joint effect from all causes (1.002, 1.000 to 1.004) were observed.

**Conclusions:**

The present findings indicated that silica exposure in combination with cigarette smoking accounted for a fraction of extra deaths in our cohort. Our research showed the urgent need for smoking cessation and silica control among iron miners.

## Background

Crystalline silica is one of the most abundant minerals in the earth’s crust and a basic component of sand, soil and granite. Environmental exposure to crystalline silica can occur not only in various natural phenomena such as volcanic explosion and sandstorms but also in agricultural areas and a series of industries, such as metal mining, pottery process, quarrying, construction, and clay manufacturing. Volcanoes and sands are major sources of silica. It has been reported that approximately 9% of the world’s population lives within 100 km of active volcanoes [1], and more than 30% of the population has been exposed to sandstorms. In industrial areas, more than 30 million workers have been exposed to silica worldwide [2, 3]. The adverse health effects of silica exposure are an increasing public health concern in recent years [4, 5]. Long-term exposure to silica dust has been confirmed to be associated with elevated mortality from silicosis, chronic obstructive pulmonary disease (COPD) [6, 7] and other airway diseases, while recent research has further revealed its association with elevated mortality from pulmonary tuberculosis [8, 9], lung cancer [10–12] and cardiovascular disease [3, 13].

Cigarette smoking is a common health problem in the world. According to the World Health Organization, China has approximately 320 million smokers, representing one third of the world’s total smokers [14]. Accumulating evidence has suggested that cigarette smoking is associated with increased mortality risks from all causes, malignant neoplasm (especially lung cancer) [15], digestive system tumours [16], ischaemic heart disease [17], and stroke [18]. Published investigations have also reported that the rate of smoking among manual workers was relatively high [19]. In our previous study [20], approximately 64% workers in metal mines and pottery factories had a history of smoking cigarettes. Based on these facts, consequences due to silica exposure in combination with cigarette smoking need to be explored in detail. However, the joint effect of silica exposure and cigarette smoking on mortality from all causes and specific diseases, especially cardiovascular disease and non-malignant respiratory disease, has not been well evaluated.

Therefore, we presented a retro-prospective cohort study of 7,665 participants from one Chinese iron mine from January 1, 1960 to December 31, 2012. For each participant, cumulative crystalline silica exposure was calculated by linking work histories with a job-exposure matrix since 1950. Cigarette smoking information was collected through questionnaires from self-report and next-of-kin (or colleagues). The objectives of this study were to assess the health effects of silica exposure and cigarette smoking on total and cause-specific mortality and to evaluate the joint effects of silica exposure and cigarette smoking on total and cause-specific mortality.

## Methods

### Study population

This retro-prospective cohort included 7,665 participants who were registered in one iron mine company located in central China and worked for at least 1 year between January 1, 1960 and December 31, 1974. This cohort is one part of the Chinese silica cohort that has been described previously [3, 21]. All participants were followed up until they died, were lost to follow-up, or survived to the end of 2012. Data on personal information and work history were retrospectively collected in 1986, and job information, vital status, cause of death and new diagnosis of pneumoconiosis were prospectively updated yearly until 2012 by industrial hygienists.

### Cause of death

Vital status was traced by local physicians during the follow-up. The cause of death for the deceased was collected from medical records in the hospital (84.45%), employment registers, accident records or death certificates in the company (15.34%), or oral reports from relatives (0.21%). The 10th version of the International Classification of Diseases (ICD-10) was used to code and classified the causes of death into different disease systems.

### Silica exposure data

Monthly measurement of dust concentration in workplaces with silica exposure was required by the Chinese government enforcement system in the early 1950s. A dust monitoring scheme including total dust concentration and crystalline silica content has been conducted by professional staff in iron mines since 1950. Respirable silica concentration in workplaces was then converted from total dust concentration and silica percent in dust particles through a field study [22, 23]. Then, a job-exposure matrix (JEM) with job- and calendar year-specific silica concentrations was established [3]. The work history for each participant including job titles and work start and end dates was taken from company occupational records. Cumulative respirable silica dust exposure (CDE) for each participant (mg/m^3^-years) was calculated as Eq. (1):

1 $$ CDE={\sum}_{i=1}^n\left({C}_i\times {T}_i\right) $$

where *n* is the total number of job titles, *C* is the 8-h-weighted mean respirable silica concentration for the ***i*th** job title, and *T* is the duration of working years for the ***i*th** job title.

## Smoking information

Detailed smoking data for all participants were first collected in 1986 and updated in 1995, 2004, and 2012. Cigarette smoking is defined as having at least 1 cigarette per day for at least 6 months. Smoking information from 728 of the participants (9.5%) was provided by their relatives because they either died or withdrew before 1986. Smoking amount was calculated by multiplying packs per day by years of smoking. To examine the reliability of the data, 385 randomly selected subjects were re-investigated in 2004. Agreement on smoking status (never or ever) between next-of-kin and colleagues of decedents was 89.1%, and agreement on smoking status between self-report and next-of-kin (or colleagues) for living subjects was 93.6% [20].

### Statistical analysis

Based on the distribution of CDE in the entire cohort, we equally divided participants into unexposed, and low, medium, and high silica exposure groups. Cox proportional hazards regression models were used for estimating hazard ratios (HRs) and 95% confidence intervals (CIs) for total and cause-specific mortality in different silica exposure levels when compared with the unexposed group. Additionally, by fitting the models with CDE as a continuous variable instead of a categorical one, we determined the correlation between HRs and per one mg/m^3^-y increase in CDE. The other covariates were adjusted, including gender, year at hire (five categories, 1955 or earlier, 1956–1960, 1961–1965, 1966–1970, and 1971 or later), age at hire (continuous) and smoking intensity (pack-years, continuous).

The participants who smoked were divided into two groups by median pack-years. We also introduced pack-year as a continuous variable to determine the correlation between HRs and per unit increase in pack-years. The other confounding factors, such as gender, year at hire (five categories, 1955 or earlier, 1956–1960, 1961–1965, 1966–1970, and 1971 or later), age at hire (continuous) and silica exposure (continuous), were adjusted.

The population attributable risks (PARs) were calculated as Eq. (2):

2 $$ PAR=\left[P\times \left( RR-1\right)\right]/\left[P\times \left( RR-1\right)+1\right] $$

where *P* is the percentage of exposure among the study population, and *RR* is the relative risk estimated from *HR*.

To assess the additive joint effects, the relative excess risks due to interaction (RERIs) needed to be evaluated. A linear relative rate model [24] was thus implemented, with the relative risks (RRs) of dichotomized silica exposure and smoking intensity at different levels calculated as Eq. (3):

3 $$ RR=\left(e\hat{\mkern6mu} {\beta}_0\right)\ast \left[1+{\beta}_1A+{\beta}_2B+{\beta}_3\left(A\ast B\right)\right] $$

where *β*_1_ and *β*_2_ is the excess relative risk per unit of exposure to A and B, respectively, and *β*_3_ is the departure from additivity of the exposure effects on an relative risk scale. Therefore, *β*_3_ is taken as estimator of RERI.

To assess the multiplicative joint effects, we constructed the Cox regression models by including continuous metrics for both smoking intensity and silica exposure as well as a product term for the interaction of them and determined the HRs. A *p*-value smaller than 0.05 was considered statistically significant. All *p*-values were 2-sided. We conducted all data analyses using SAS, version 9.3 software (SAS Inc. Cary, North Carolina).

## Results

A total of 7,665 participants (6,542 male, 85.4%) with median follow-up of 42.8 years were included into analyses. The average participant age upon entry to the cohort was 24.8 years. At the end of follow-up, only 38 (0.5%) participants were still employed, while 6,519 (85.0%) participants had retired or died, and 1,108 (14.5%) had left for other companies. Among those who left for other companies, 270 individuals were lost to follow-up, and their person-years were thus truncated at time of loss.

Table 1 shows characteristics of the cohort according to different silica exposure levels. A total of 3,658 (47.7%) participants were exposed to silica dust during their working period, and 96.2% of silica-exposed workers were male. The number of smokers (current and former) among all participants and silica-exposed workers were 5,070 (66.1%) and 2,732 (74.7%), respectively. The percentage of smokers was 76.9% for male workers and 3.3% for female workers. During the follow-up, 2,814 deaths were identified during 315,772.9 person-years of follow-up. Mortality was 891.2 per 100,000 person-years for all participants. The mortality was 1042.0 per 100,000 person-years and 751.6 per 100,000 person-years among silica-exposed and non-silica-exposed workers, respectively. Among the deaths, 1,581 (56.2%) were silica-exposed workers, and 1,917 (68.1%) were smokers.

**Table 1:** Basic characteristics of participants (*n* = 7,665) by silica exposure level, 1960–2012

Characteristic	Entire cohort (*n* = 7,665)	Silica exposure level^a^			
		Unexposed (*n* = 4,007)	Low (*n* = 1,218)	Medium (*n* = 1,220)	High (*n* = 1,220)
Male gender (*n*, %)	6542 (85.35)	3023 (75.44)	1160 (95.24)	1181 (96.80)	1178 (96.56)
Year at birth	1939.50 ± 9.60	1940.23 ± 9.48	1943.15 ± 9.08	1938.77 ± 9.40	1934.15 ± 8.29
Year at hire	1962.28 ± 7.06	1963.01 ± 6.83	1965.87 ± 6.17	1962.06 ± 6.19	1956.51 ± 5.94
Age at hire (years)	22.78 ± 5.76	22.78 ± 5.89	22.72 ± 5.57	23.29 ± 5.92	22.36 ± 5.30
Ever smoker (*n*, %)^b^	5070 (66.14)	2338 (58.35)	893 (73.32)	926 (75.90)	913 (74.84)
Never smoker (*n*, %)^b^	2595 (33.86)	1669 (41.65)	325 (26.68)	294 (24.10)	307 (25.16)
Pack-years of ever smoker ^b^	28.57 ± 15.39	28.27 ± 14.95	26.52 ± 14.11	29.03 ± 16.74	30.86 ± 15.95
Age at first exposure (years)^c^	23.65 ± 6.36	NA	24.54 ± 7.27	23.79 ± 6.09	22.63 ± 5.44
Cumulative total dust concentration (mg/m^3^-y)^c^	117.95 ± 70.27	NA	55.21 ± 21.94	107.32 ± 18.41	191.22 ± 67.66
Average total dust concentration (mg/m^3^)^c^	5.29 ± 3.02	NA	3.88 ± 2.49	4.66 ± 2.05	7.34 ± 3.22
Cumulative respirable dust concentration (mg/m^3^-y)^c^	18.48 ± 11.01	NA	10.34 ± 6.53	18.02 ± 7.03	27.08 ± 11.46
Average respirable dust concentration (mg/m^3^)^c^	0.83 ± 0.47	NA	0.69 ± 0.47	0.77 ± 0.37	1.03 ± 0.50
Cumulative respirable silica dust concentration (mg/m^3^-y)^c^	0.83 ± 0.80	NA	0.34 ± 0.13	0.66 ± 0.10	1.51 ± 1.07
Average respirable silica dust concentration (mg/m^3^)^c^	0.04 ± 0.04	NA	0.02 ± 0.02	0.03 ± 0.01	0.06 ± 0.06
Duration of dust exposure (years)^c^	23.95 ± 9.29	NA	18.12 ± 9.65	25.55 ± 7.13	28.17 ± 7.80
Pneumoconiosis cases (*n*, %)^d^	332 (9.08)	NA	21 (1.72)	79 (6.48)	232 (19.02)
Age of first diagnosis of pneumoconiosis (years)^d^	49.64 ± 8.83	NA	51.11 ± 12.88	50.84 ± 9.04	49.11 ± 8.29
Latency of pneumoconiosis (years)^d^	25.60 ± 8.00	NA	23.67 ± 9.32	24.69 ± 7.69	26.08 ± 7.96
Age upon entry to cohort (years)	24.84 ± 6.73	24.39 ± 6.61	23.63 ± 6.18	25.05 ± 6.67	27.34 ± 7.09
Median of follow-up (years)^e^	42.75 ± 12.00	42.34 ± 14.00	41.92 ± 5.92	43.09 ± 10.04	45.34 ± 14.33
Deceased (*n*, %)	2814 (36.71)	1233 (30.77)	401 (32.92)	515 (42.21)	665 (54.51)

Values were generally presented as mean ± standard deviation, if not specially indicated

^a^Silica exposure was divided into four levels, i.e. unexposed, low (0.4935 mg/m^3^-y or below), medium (0.4935 to 0.8423 mg/m^3^-y), and high level(0.8423 mg/m^3^-y or above)

^b^Cigarette smoking was defined as taking at least 1 cigarette per day for at least 6 months, and pack-years were calculated only among ever smokers

^c^Exposure-related results were figured out only among dust-exposed workers

^d^Pneumoconiosis-related results were figured out only among workers diagnosed with pneumoconiosis. Latency of pneumoconiosis was defined as the duration from the onset of dust exposure to the first diagnostic time of pneumoconiosis

^e^Values hereby were presented as median ± inter quartile range

NA, i.e. the unavailable values

**Fig. 1:**
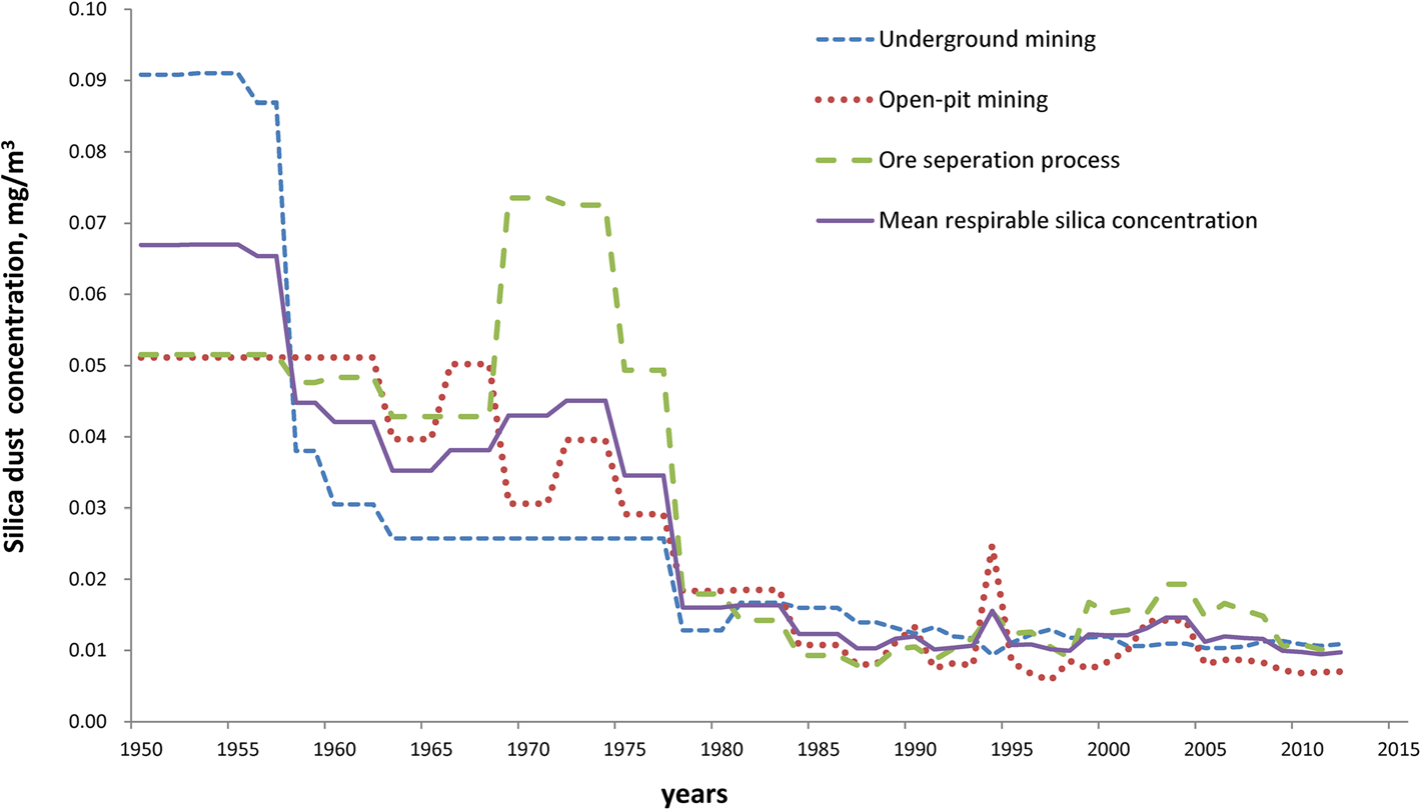
Annual respirable silica concentration for different job titles, classified by major activity groups, 1950–2012. Horizontal axis represents the year of industrial production and vertical axis represents respirable silica concentration. Solid line represents annual average respirable silica concentration among all the silica-exposed job titles and the other three types of dotted lines represent annual average respirable silica concentration among the specific major activity groups, that is underground mining, open-pit mining and ore separation process group

Annual respirable silica concentrations for different job titles classified by major activity groups from 1950 to 2012 are listed in Fig. 1. The mean respirable silica concentrations ranged from 0.03 mg/m^3^ to 0.07 mg/m^3^ before 1978. After taking protection measures, silica concentration fell to between 0.01 mg/m^3^ and 0.02 mg/m^3^ from 1978 to 2010 and gradually decreased to below 0.01 mg/m^3^ in 2012. The silica concentration for the underground mining group was higher than 0.09 mg/m^3^ in the early 1950s and then decreased stably. The silica concentration for the ore separation process group was approximately 0.05 mg/m^3^ before 1969 and became higher than 0.07 mg/m^3^ between 1970 and 1974 due to adoption of mechanical crushing, and then the concentration decreased to lower than 0.02 mg/m^3^ after 1975 due to ventilation and wet work.

Table 2 shows the number of deaths and HRs for total and cause-specific mortality due to silica exposure. Malignant neoplasm was the leading cause of death in the iron mine. The second to fourth causes of death in this cohort were cerebrovascular disease, cardiovascular disease, and non-malignant respiratory disease, respectively. Compared with the unexposed group, significantly higher mortality was found in silica-exposed workers from all causes (HR 1.303, 95% CI 1.206 to 1.408), malignant neoplasm (1.357, 1.173 to 1.570), cardiovascular disease (1.446, 1.169 to 1.789) and non-malignant respiratory disease (3.166, 2.440 to 4.107). A positive exposure-response relationship was found between (both categorical and continuous) CDE and mortality from all causes, non-malignant respiratory disease (including pneumoconiosis), cardiovascular disease (including pulmonary heart disease), certain infectious and parasitic diseases (including respiratory tuberculosis), and lung cancer. Each 1 mg/m^3^-y increase in CDE was associated with an 11.7, 41.4, 21.3 and 19.0% increase in mortality risk from all causes, non-malignant respiratory disease, cardiovascular disease, and lung cancer, respectively. In addition, a positive exposure-response relationship existed between categorical CDE and mortality from ischaemic heart disease, and the HRs were 1.190, 1.538 and 1.781 (monotonically increased) for low, medium and high levels of CDE (*P*-value for linear trend: < 0.001), respectively, although the relationship between continuous CDE and mortality was not statistically clear (HR 1.134, 0.990 to 1.298).

**Table 2:** HRs for total and cause-specific mortality associated with silica exposure in the cohort (*n* = 7,665), 1960–2012

Cause of death (ICD-10)	Number of deaths	HRs for per 1 mg/m^3^-y increase in CDE	HRs for silica exposure levels versus unexposed^a^			
			Low	Medium	High	*p*-Value^b^
Malignant neoplasm (C00-C97)	789	1.048 (0.962, 1.142)	1.531 (1.241, 1.890)	1.378 (1.137, 1.669)	1.238 (1.025, 1.494)	0.009
Malignant neoplasm of oesophagus (C15)	66	0.952 (0.693, 1.307)	1.335 (0.648, 2.753)	1.511 (0.795, 2.874)	1.249 (0.644, 2.420)	0.384
Malignant neoplasm of stomach (C16)	104	1.130 (0.931, 1.373)	1.980 (1.153, 3.401)	1.380 (0.800, 2.379)	1.367 (0.814, 2.295)	0.223
Malignant neoplasm of liver and intrahepatic bile ducts (C22)	152	0.774 (0.585, 1.025)	1.512 (0.972, 2.353)	1.060 (0.681, 1.648)	0.774 (0.487, 1.229)	0.359
Lung cancer (C33-C34)	262	1.190 (1.049, 1.349)	1.671 (1.132, 2.466)	1.663 (1.193, 2.318)	1.671 (1.216, 2.298)	0.001
Certain infectious and parasitic diseases (A00-B99, J65)	89	1.204 (1.018, 1.423)	1.073 (0.520, 2.212)	1.560 (0.882, 2.761)	1.790 (1.064, 3.010)	0.020
Respiratory tuberculosis (A15-A16, J65)	58	1.226 (1.017, 1.477)	0.523 (0.153, 1.785)	1.459 (0.695, 3.061)	2.532 (1.383, 4.635)	0.002
Cardiovascular disease (I00-I52, I70-I99)	372	1.213 (1.117, 1.318)	0.986 (0.672, 1.447)	1.373 (1.030, 1.831)	1.723 (1.345, 2.206)	< 0.001
Ischaemic heart disease (I20-I25)	219	1.134 (0.990, 1.298)	1.190 (0.738, 1.918)	1.538 (1.063, 2.226)	1.781 (1.281, 2.476)	< 0.001
Pulmonary heart disease (I26-I27)	66	1.352 (1.197, 1.529)	0.562 (0.168, 1.880)	1.258 (0.615, 2.573)	2.009 (1.160, 3.479)	0.010
Cerebrovascular disease (I60-I69)	388	0.973 (0.859, 1.103)	0.950 (0.670, 1.345)	1.234 (0.945, 1.613)	0.945 (0.724, 1.233)	0.995
Disease of the respiratory system (J00-J99)	324	1.414 (1.329, 1.505)	2.817 (1.927, 4.118)	2.714 (1.954, 3.768)	3.615 (2.718, 4.807)	< 0.001
Pneumoconiosis (J60-J65)	110	1.384 (1.255, 1.525)	1.000 (reference)	2.245 (0.912, 5.524)	5.312 (2.298, 12.280)	< 0.001
Chronic lower respiratory disease (J40-J47)	142	1.264 (1.115, 1.434)	2.759 (1.688, 4.507)	2.060 (1.304, 3.253)	1.720 (1.106, 2.675)	0.012
Disease of the digestive system (K00-K93)	95	0.877 (0.635, 1.211)	1.467 (0.831, 2.587)	1.065 (0.605, 1.874)	0.838 (0.468, 1.499)	0.629
Fibrosis and cirrhosis of liver (K74)	63	0.770 (0.487, 1.218)	1.302 (0.651, 2.601)	1.079 (0.549, 2.121)	0.714 (0.335, 1.524)	0.488
External causes of morbidity and mortality (V01-Y98)	193	0.883 (0.702, 1.110)	2.839 (1.996, 4.038)	1.145 (0.745, 1.761)	0.928 (0.591, 1.458)	0.953
All diseases (A00-Y98)	2814	1.117 (1.074, 1.162)	1.368 (1.218, 1.537)	1.246 (1.121, 1.383)	1.313 (1.192, 1.447)	< 0.001

HRs were calculated by using Cox proportional hazards models, which included the duration of follow-up as time variable and were adjusted for gender, year at hire (five categories: 1955 or earlier, 1956–1960, 1961–1965, 1966–1970, 1971 or later), age at hire (continuous), and smoking intensity (continuous). Pneumoconiosis mortality risks were estimated only among silica-exposed workers

^a^The categorical analyses were conducted on the basis of various silica exposure level, i.e. unexposed, low (0.4935 mg/m^3^-y or below), medium (0.4935 to 0.8423 mg/m^3^-y), and high level (0.8423 mg/m^3^-y or above). The reference category was defined as the unexposed level, except pneumoconiosis, which used low level as reference category

^b^Evaluated by including the median exposure values within each exposure level as a continuous variable in the model

Table 3 shows the death number of ever and never smokers and HRs for total and cause-specific mortality due to cigarette smoking. As seen, malignant neoplasm had the largest proportion of ever smokers (77.95%). When compared with the never smokers, significantly increased mortality risks were found from all causes (HR 1.102, 95% CI 1.008 to 1.205), non-malignant respiratory disease (1.612, 1.229 to 2.115), pulmonary heart disease (2.067, 1.112 to 3.843), malignant neoplasm (1.694, 1.404 to 2.045) and lung cancer (4.347, 2.820 to 6.700) among the smokers, while the increment was not significant from ischaemic heart disease (1.126, 0.823 to 1.539). A statistically significant exposure-response relationship was found between smoking amount (both categorical and continuous) and mortality from malignant neoplasm (including lung cancer) and non-malignant respiratory disease (including chronic lower respiratory disease). Each 1 pack-year increase in smoking was associated with a 0.7, 1.0, 0.7 and 2.3% increase in the mortality risk from non-malignant respiratory disease, chronic lower respiratory disease, malignant neoplasm and lung cancer, respectively. Furthermore, we found a positive exposure-response relationship between categorical smoking and mortality from pneumoconiosis with a linear *p*-trend of 0.005, despite of the fact that the HR for each 1 pack-year increase in smoking did not reach statistical significance (HR 1.008, 0.999 to 1.017).

**Table 3:** HRs for total and cause-specific mortality associated with cigarette smoking in the cohort (n = 7,665), 1960–2012

Cause of death (ICD-10)	Ever/Never smokers	HR for per 1 pack-year increase in smoking	HRs for smoking intensity versus non-smoking^a^		
			< 26.08 pack-years	≥26.08 pack-years	*p*-trend^b^
Malignant neoplasm (C00-C97)	615/174	1.007 (1.004, 1.011)	1.689 (1.364, 2.090)	1.698 (1.392, 2.072)	< 0.001
Malignant neoplasm of oesophagus (C15)	46/20	0.997 (0.984, 1.010)	0.944 (0.487, 1.827)	0.979 (0.535, 1.790)	0.956
Malignant neoplasm of stomach (C16)	72/32	0.990 (0.979, 1.001)	1.207 (0.746, 1.952)	0.617 (0.372, 1.022)	0.050
Malignant neoplasm of liver and intrahepatic bile ducts (C22)	120/32	0.991 (0.982, 1.000)	2.142 (1.379, 3.326)	0.968 (0.609, 1.538)	0.511
Lung cancer (C33-C34)	228/34	1.023 (1.019, 1.028)	1.883 (1.111, 3.192)	6.115 (3.908, 9.570)	< 0.001
Certain infectious and parasitic diseases (A00-B99, J65)	62/27	0.982 (0.970, 0.995)	1.504 (0.893, 2.533)	0.633 (0.358, 1.122)	0.097
Respiratory tuberculosis (A15-A16, J65)	43/15	0.991 (0.976, 1.005)	1.756 (0.889, 3.471)	1.009 (0.503, 2.025)	0.905
Cardiovascular disease (I00-I52, I70-I99)	259/113	1.002 (0.997, 1.007)	1.546 (1.178, 2.029)	1.012 (0.774, 1.324)	0.885
Ischaemic heart disease (I20-I25)	150/69	1.005 (0.998, 1.011)	1.173 (0.810, 1.699)	1.097 (0.782, 1.539)	0.632
Pulmonary heart disease (I26-I27)	51/15	1.007 (0.995, 1.019)	3.097 (1.608, 5.965)	1.365 (0.675, 2.760)	0.507
Cerebrovascular disease (I60-I69)	258/130	1.000 (0.995, 1.005)	1.054 (0.801, 1.389)	0.973 (0.757, 1.249)	0.797
Disease of the respiratory system (J00-J99)	246/78	1.007 (1.002, 1.012)	1.473 (1.068, 2.031)	1.701 (1.275, 2.268)	< 0.001
Pneumoconiosis (J60-J65)	93/17	1.008 (0.999, 1.017)	2.928 (1.638, 5.237)	2.458 (1.394, 4.334)	0.005
Chronic lower respiratory disease (J40-J47)	104/38	1.010 (1.002, 1.017)	1.058 (0.633, 1.768)	1.745 (1.143, 2.665)	0.006
Disease of the digestive system (K00-K93)	63/32	0.978 (0.966, 0.990)	1.369 (0.843, 2.223)	0.380 (0.212, 0.684)	0.001
Fibrosis and cirrhosis of liver (K74)	42/21	0.975 (0.960, 0.991)	1.305 (0.721, 2.362)	0.360 (0.174, 0.748)	0.006
External causes of morbidity and mortality (V01-Y98)	120/73	0.960 (0.950, 0.970)	0.980 (0.706, 1.360)	0.262 (0.170, 0.405)	< 0.001
All diseases (A00-Y98)	1917/897	1.001 (0.999, 1.003)	1.176 (1.060, 1.305)	1.056 (0.958, 1.163)	0.413

HRs were calculated by using Cox proportional hazards models, which included the duration of follow-up as time variable and were adjusted for gender, year at hire (five categories: 1955 or earlier, 1956–1960, 1961–1965, 1966–1970, 1971 or later), age at hire (continuous), and silica exposure (pack-years, continuous). Pneumoconiosis mortality risks were estimated only among silica-exposed workers

^a^The categorical analyses were conducted on the basis of smoking intensity, including non-smoking, < 26.08 pack-years and ≥ 26.08 pack-years, with 26.08 pack-years as the median point for smoking intensity. The reference category was defined as the non-smoking level

^b^Evaluated by including the median exposure values within each smoking intensity category as a continuous variable in the model

After adjustment for potential confounders and smoking, we found that silica exposure accounted for 12.6, 50.8, 17.5 and 24.2% of mortality from all deaths, non-malignant respiratory disease, cardiovascular disease, and lung cancer, respectively. Likewise, after adjustment for potential confounders and silica exposure, cigarette smoking accounted for 6.3, 28.8, 12.6 and 68.9% of mortality from all deaths, non-malignant respiratory disease, cardiovascular disease, and lung cancer, respectively. Eventually, combined exposure to silica dust and cigarette smoking accounted for 21.2, 76.0, 35.7 and 81.4% of mortality from all deaths, non-malignant respiratory disease, cardiovascular disease, and lung cancer, respectively.

When compared with non-silica-exposed and non-smoking workers, we found significantly elevated mortality risks from all causes (HR 1.344, 95% CI 1.189 to 1.520), non-malignant respiratory disease (5.052, 3.144 to 8.116), cardiovascular disease (1.709, 1.214 to 2.405) and lung cancer (6.586, 3.616 to 11.992) among silica-exposed and smoking workers. The joint effects of silica exposure and cigarette smoking on total and cause-specific mortality are shown in Table 4. From combined effects analysis, the relative excess risks due to interaction (RERIs) of smoking and silica exposure were 1.893 (95% CI 0.628 to 3.441) for lung cancer and 6.457 (0.725 to 39.114) for pneumoconiosis, indicating that joint effects were significant and more than additive. The additive joint effects could be further observed without significance for all causes, malignant neoplasm, and non-malignant respiratory disease. A multiplicative interaction between silica exposure and smoking was only significant for all causes (1.002, 1.000 to 1.004).

**Table 4:** Estimated HRs for mortality, associated with combined exposure to silica and smoking (n = 7,665), 1960–2012

Cause of death (ICD-10)	Hazard ratio and 95% confidence interval		Interaction effect for additivity (RERI)^b^	Interaction factor on multiplicative scale^b^
	Unexposed workers	Exposed workers		
Malignant neoplasm (C00-C97)				
Never smokers	1.000 (reference)	1.271 (0.923, 1.748)	0.306 (− 0.306, 0.835)	0.999 (0.995, 1.004)
Ever smokers	1.602 (1.212, 2.119)	2.196 (1.679, 2.871)		
Lung cancer (C33-C34)				
Never smokers	1.000 (reference)	1.460 (0.698, 3.051)	1.893 (0.628, 3.441)	0.999 (0.993, 1.005)
Ever smokers	3.840 (2.079, 7.094)	6.586 (3.616, 11.992)		
Cardiovascular disease (I00-I52, I70-I99)				
Never smokers	1.000 (reference)	1.445 (0.979, 2.132)	−0.237 (− 1.062, 0.381)	1.002 (0.997, 1.007)
Ever smokers	1.190 (0.828, 1.711)	1.709 (1.214, 2.405)		
Ischaemic heart disease (I20-I25)				
Never smokers	1.000 (reference)	1.487 (0.900, 2.455)	−0.144 (− 1.196, 0.592)	1.005 (0.998, 1.013)
Ever smokers	1.049 (0.651, 1.690)	1.690 (1.083, 2.635)		
Disease of the respiratory system (J00-J99)				
Never smokers	1.000 (reference)	3.488 (2.095, 5.808)	0.136 (− 1.784, 1.633)	1.002 (0.998, 1.006)
Ever smokers	1.679 (0.996, 2.828)	5.052 (3.144, 8.116)		
Pneumoconiosis (J60-J65)^a^				
Never smokers	1.000 (reference)	3.319 (0.757, 14.560)	6.457 (0.725, 39.114)	1.003 (0.996, 1.009)
Ever smokers	1.973 (0.429, 9.067)	8.658 (2.110, 35.530)		
Chronic lower respiratory disease (J40-J47)				
Never smokers	1.000 (reference)	3.200 (1.566, 6.536)	−1.170 (− 4.236, 0.488)	1.000 (0.993, 1.008)
Ever smokers	2.142 (1.057, 4.341)	3.725 (1.901, 7.301)		
All diseases (A00-Y98)				
Never smokers	1.000 (reference)	1.139 (0.989, 1.313)	0.158 (−0.136, 0.421)	1.002 (1.000, 1.004)
Ever smokers	0.977 (0.858, 1.113)	1.344 (1.189, 1.520)		

HRs were calculated by using Cox proportional hazards models, which included the durations of follow-up as time variables and were adjusted for gender, year at hire (five categories: 1955 or earlier, 1956–1960, 1961–1965, 1966–1970, 1971 or later), age at hire (continuous)

The dichotomous analyses were conducted on the basis of silica exposure (unexposed or exposed) and smoking status (never of ever), and the reference category was defined as the non-silica-exposed and non-smoking level, except pneumoconiosis

^a^For pneumoconiosis, the dichotomous analysis was conducted on the basis of silica exposure (lower or higher level divided by 0.65328 mg/m^3^-y) and smoking status (never of ever), and the reference category was defined as the lower-silica-exposed and non-smoking level

^b^Interaction effects were not statistically significant, unless 0 was included into the confidence intervals of relative excess risk due to interaction (RERI), or 1 was included into the confidence intervals of interaction factor on multiplicative scales

## Discussion

In our cohort study of 7,665 workers from one Chinese iron mine, we not only confirmed an exposure-response relationship between long-term exposure to cumulative crystalline silica dust and mortality risk from all causes, non-malignant respiratory disease, cardiovascular disease and lung cancer but also found significant exposure-response relationships between lifelong cigarette smoking and mortality from non-malignant respiratory disease and lung cancer. Furthermore, we observed significant additive joint effects of silica exposure and cigarette smoking on mortality from lung cancer and pneumoconiosis, together with a significant multiplicative joint effect from all causes.

A significant positive exposure-response relationship was found between CDE and mortality from all causes, non-malignant respiratory disease, respiratory tuberculosis, and cardiovascular disease among 74,040 Chinese workers in our previous analysis [3]. In the present study, we extended 10 years of follow-up for all participants from one iron mine of the above cohort. At the end of follow-up, only 0.5% participants of this cohort were still working. In other words, we completed the entire follow-up of lifetime occupational exposure to silica dust for more than 99% participants and could therefore provide reliable evaluations about adverse health effects from silica exposure. Our cohort reconfirmed an obvious exposure-response relationship between silica exposure and mortality from all causes, non-malignant respiratory disease, cardiovascular disease, and lung cancer.

Consistent with our study, research at an iron ore mine in Cumbria showed significantly elevated standardized mortality ratios (SMRs) from all causes (1.27), neoplasm (1.18), ischaemic heart disease (1.18), and respiratory disease (1.73) [25]. Another iron ore mine cohort study in Sweden with 5 years of follow-up reported significantly elevated mortality risks from all causes (SMR 1.05, 95% CI 1.02 to 1.09), lung cancer (1.73, 1.52 to 1.97), respiratory disease (1.14, 1.00 to 1.28), and myocardial infarction (1.12, 1.07 to 1.18) [26]. However, a retrospective research from a Minnesota haematite cohort did not find any excess death rates from all causes or respiratory disease, which might be due to insufficient control for smoking or potential interference from other unknown types of productive dusts [27].

As a serious public health concern, smoking contributes to increased mortality from cardiovascular disease, respiratory disease and malignant neoplasm. Our study strongly demonstrated the exposure-response relationship between smoking amount and mortality from non-malignant respiratory disease and lung cancer, two main causes of death occupying the top rank in much of the current smoking research worldwide. The World Health Organization estimated that approximately 100 million people died of smoking across the twentieth century [28]. In addition, according to the Surgeon General’s Report in 2014 [29], smoking causes one of every three deaths from cardiovascular disease. In our study, an increase in mortality from cardiovascular disease (including ischaemic and pulmonary heart disease) was also observed among smokers when compared with non-smokers.

A large amount of research has testified to the influence of smoking on mortality. According to 50 years of observational research on British male doctors, the excess mortality associated with cigarette smoking was elevated for men born before 1900 (SMR = 1.16) and peaked for men born in the 1920s (SMR = 2.83). The probabilities of dying in middle age were 42 and 24% among smokers and non-smokers (a 2-fold mortality ratio) for those born in the 1910s, and 43% vs 15% (a 3-fold mortality ratio) for those born in the 1920s, respectively [30]. A cohort study in New Zealand also reported that the relative effect of smoking on mortality differed over time [31]. Additionally, a pooled study provided 2-fold and 1.3-fold excess mortality for current and former smokers compared with those who never smoked, and the excess mortality decreased with time since cessation of smoking [32].

Smoking acts as an essential inducing factor for lung cancer and non-malignant respiratory disease on its own, just as our research suggested. In addition, smoking, especially cigarette smoking, modifies the relationship between silica exposure and mortality from lung cancer and non-malignant respiratory disease. Because of the inherent toxicity and carcinogenicity of cigarettes [33], the adverse health effects due to combined exposure to silica dust and cigarette smoking are strengthened to some extent. In our previous research with 34,018 workers over 44 years of follow-up [20], a joint effect of occupational silica exposure and cigarette smoking on mortality from lung cancer was discovered to be greater than additive models (RERI 0.98, 0.23 to 1.74) and very close to multiplicative models (interaction term 1.27, 0.75 to 2.15). Similar results were also reported from a pooled analysis in Canada, which discovered that joint effects of silica exposure and smoking were between additive and multiplicative models, perhaps closer to the latter [34]. Our present study provided obvious results again for lung cancer, confirming a joint effect far beyond additive models (RERI 1.893, 0.628 to 3.441) and approaching multiplicative models (interaction term 0.999, 0.993 to 1.005). The significant additive joint effect of silica and cigarettes for lung cancer can be explained by the synergy of cigarette-induced genetic and cellular toxicity [33] and the silica-induced pulmonary inflammation and fibrosis [35].

Further, our research evaluated potential joint effects of silica exposure and smoking on mortality from other diseases. Significant additive interactions were observed on the mortality from lung cancer and pneumoconiosis, and a significant multiplicative interaction was found on the mortality from all causes. The analysis of population attributable risks (PARs) provided similar results, where combined exposure to silica dust and cigarette smoking accounted for elevated proportions of mortality from all causes, non-malignant respiratory disease, cardiovascular disease, and lung cancer compared with exposure to silica dust or cigarette smoking alone. Published literature has also suggested that persistent inflammation induced by silica dust may be related to health damage not only from lungs [36] but also from kidney, heart and liver [5]. On the other hand, cigarette smoking may exacerbate persistent toxicity and inflammation induced by silica exposure.

This study has several advantages. First, the cohort is large with a long period of follow-up and a low rate of withdrawal. Second, detailed data on dust concentration from the workplace and personal work history were acquired to estimate cumulative respirable silica exposure for each participant. Moreover, we have collected detailed and accurate smoking data for each participant.

One limitation of this study is that the silica concentration before 1950 was estimated using the concentration in 1950, which might lead to underestimated exposure for workers who started working before 1950 (140 workers). However, the results did not change after excluding the participants who started work before 1950. Another limitation is that cigarette smoking data for the deceased derived from their colleagues or next-of-kin, and recall bias might exist as a consequence. Lastly, the healthy worker survivor effect might underestimate risk of mortality from total and specific causes. Nevertheless, the results did not change substantially when we put the duration of employment into the regression models to re-analyse mortality risks with regard to silica, smoking and their combined effects.

## Conclusions

In this cohort study of an iron mine, we confirmed adverse health effects of long-term crystalline silica exposure and life-long cigarette smoking. The joint effects of silica exposure and cigarette smoking were found on mortality from lung cancer and all causes, which indicate that silica in combination with cigarette smoking accounts for a fraction of extra deaths. Therefore, more effective measures should be taken towards silica control and smoking cessation among iron mine workers.

## Abbreviations

CDE: Cumulative respirable silica dust exposure
CI: Confidence interval
COPD: Chronic obstructive pulmonary disease
HR: Hazard ratio
ICD-10: The 10th version of the international classification of diseases
JEM: Job-exposure matrix
PAR: Population attributable risk
RERI: Relative excess risk due to interaction
SMR: Standardized mortality ratio
